# Reconstitution of *Drosophila* and human chromatins by wheat germ cell-free co-expression system

**DOI:** 10.1186/s12896-020-00655-6

**Published:** 2020-12-01

**Authors:** Kei-ichi Okimune, Szilvia K. Nagy, Shogo Hataya, Yaeta Endo, Taichi E. Takasuka

**Affiliations:** 1 Research Faculty of Agriculture, Hokkaido University, Sapporo, Japan; 2 Graduate School of Global Food Resources, Hokkaido University, Sapporo, Japan; 3 Department of Molecular Biology, Institute of Biochemistry and Molecular Biology, Semmelweis University, Budapest, Hungary; 4 Proteo-Science Center of Ehime University, Matsuyama, Japan; 5 GI-CORE, Hokkaido University, Sapporo, 060-8589 Japan

**Keywords:** In vitro chromatin assembly, Histone, Wheat germ cell-free protein expression, Co-expression chromatin assembly, *Drosophila* chromatin, Human chromatin

## Abstract

**Background:**

Elaboration of the epigenetic regulation of chromatin is a long-standing aim in molecular and cellular biology. Hence, there is a great demand for the development of in vitro methods to reconstitute chromatin that can be used directly for biochemical assays. The widely used wheat germ cell-free protein expression method provides broad applications to investigate the function and structure of eukaryotic proteins. Such advantages, including high translation efficiency, flexibility, and possible automatization, are beneficial for achieving native-like chromatin substrates for in vitro studies.

**Results:**

We describe a novel, single-step in vitro chromatin assembly method by using the wheat germ cell-free protein synthesis. We demonstrated that both *Drosophila* and human chromatins can be reconstituted in the course of the in vitro translation of core histones by the addition of chromatin assembly factors, circular plasmid, and topoisomerase I in an ATP-dependent manner. *Drosophila* chromatin assembly was performed in 4 h at 26 °C, in the presence of premixed mRNAs encoding the core histones, dAcf1/dISWI chromatin remodeling complex, and nucleosome assembly protein, dNAP1. Similarly, the human chromatin was assembled by co-expressing the human core histones with *Drosophila* chromatin remodeling factor, dISWI, and chromatin chaperone, dNLP, for 6 h at 26 °C. The presence of reconstituted chromatin was monitored by DNA supercoiling assay, also the regular spacing of nucleosomes was assessed by Micrococcal nuclease assay. Furthermore, *Drosophila* linker histone H1-containing chromatin was reconstituted, affirming that the in vitro assembled chromatin is suitable for downstream applications.

**Conclusions:**

The method described in this study allows the assembly of *Drosophila* and human chromatins, possibly in native-like form, by using a wheat germ cell-free protein expression. Although both chromatins were reconstituted successfully, there were unexpected differences with respect to the required ratio of histone-coding mRNAs and the reaction time. Overall, our new in vitro chromatin reconstitution method will aid to characterize the unrevealed structure, function, and regulation of chromatin dynamics.

**Supplementary Information:**

**Supplementary information** accompanies this paper at 10.1186/s12896-020-00655-6.

## Background

Chromatin enables high-level compaction of the enormous genomic DNA into a few microns of nuclei in every eukaryote. The histone octamer, that is composed of four core histones, H2A, H2B, H3, and H4, is tightly wrapped around by approximately 147 base pair of DNA in a left-handed superhelix [1–4]. Moreover, spatiotemporal changes of chromatin post-translational modification and its overall structure during the cell-cycle contribute to multiple cellular processes such as DNA replication [5, 6], repair [7, 8], gene regulation [9–11], and cell proliferation [12, 13]. Such chromatin dynamics have been studied for decades, and chemical modifications on the N-terminal regions of histones are thought to play pivotal roles in the prior processes.

The first detailed nucleosome structure was described by determining the X-ray crystallography of the refolded, purified, and in vitro assembled nucleosome on human α-satellite DNA [3]. Since then, various nucleosome structures from different organisms were determined at higher resolution by X-ray crystallography and other methods [4, 14], additionally, oligomeric nucleosomal structures were obtained by cryo-electron microscopy [15]. The majority of the abovementioned approaches utilize unmodified/purified/refolded four core histones with defined DNA sequences that possess nucleosome positioning capability, and the assembly was achieved by the salt gradient method [16–18]. Based on these determined structures, the influence of particular post-translational modification of histone tails on nucleosome has been widely studied. For instance, the partially synthetic histone H4 K16Ac along with the other three core histones was assembled into nucleosome in vitro, and the physicochemical characterization of the modified nucleosome was determined [19]. Additionally, the detailed analysis of the effect of the fully chemical synthetic K56 acetylated histone H3 on nucleosome formation was reported [20]. Furthermore, similar approaches were taken place to understand the role of post-translational modifications of histone proteins on the chromatin structure and functions [21, 22].

Besides the salt dialysis chromatin reconstitution technique, ATP-dependent chromatin assembly methods have been applied in a vast range of chromatin research, which utilizes a set of chromatin assembly factors [23–26]. The first report was published by using the *Xenopus laevis* oocytes extract, and the assembly of nucleosome was determined to be ATP-dependent [27]. Then, Becker and Wu published the method by using a *Drosophila melanogaster* embryo cell-free system, and it was shown that unknown factors in the embryo facilitated the formation of chromatin [23]. Later, several key factors in the *Drosophila* embryo were defined, including ATP-utilizing assembly factor (dAcf1 and dISWI), and nucleosome assembly protein-1 (dNAP1), that together help to assemble the regularly spaced chromatin, and became one of the standard methods [25, 26, 28]. The ATP-dependent chromatin assembly methods have been often employed to investigate the molecular mechanisms of the way of the chromatin assembly in the cell nuclei since this chromatin reconstitution procedure is more analogous to the physiological process, compared to the abovementioned salt dialysis method. Recently, Kadonaga’s group reported that by using bacterially produced chromatin assembly factors, dISWI and dNLP, human chromatin reconstitution can be attained in vitro [29].

In this study, we sought to develop a novel chromatin reconstitution method by using a well-established wheat germ cell-free protein synthesis [30, 31] and on the basis of conventional chromatin assembly methods [25, 26, 28, 29]. The wheat germ cell-free protein synthesis is known to enable the production of N-methionine processed, native, and functional protein, thus making it the ideal method of choice for chromatin reconstitution [32, 33]. Previously, we and others successfully synthesized dozens of proteins of different organisms by using the wheat cell-free system [34–39], thus we applied this method for the expression of the *Drosophila* and human core histones, ATP-utilizing assembly factor, nucleosome assembly protein, and histone chaperone. For achieving the reconstitution of both *Drosophila* and human chromatins in co-expression manner, each set of core histones was translated along with the appropriate pairs of *Drosophila* chromatin assembly factors. Furthermore, the ratio of the mRNAs of core histones and incubation time were optimized in both *Drosophila* and human chromatin assembly systems. Finally, the reconstituted chromatins were evaluated by supercoiling and Micrococcal nuclease (MNase) assays. Overall, we describe a single-step in vitro chromatin assembly method, which may serve as a new, viable protocol for chromatin research and epigenetic studies.

## Results

### Histones and chromatin assembly factors synthesized by wheat germ cell-free synthesis

**Fig. 1:**
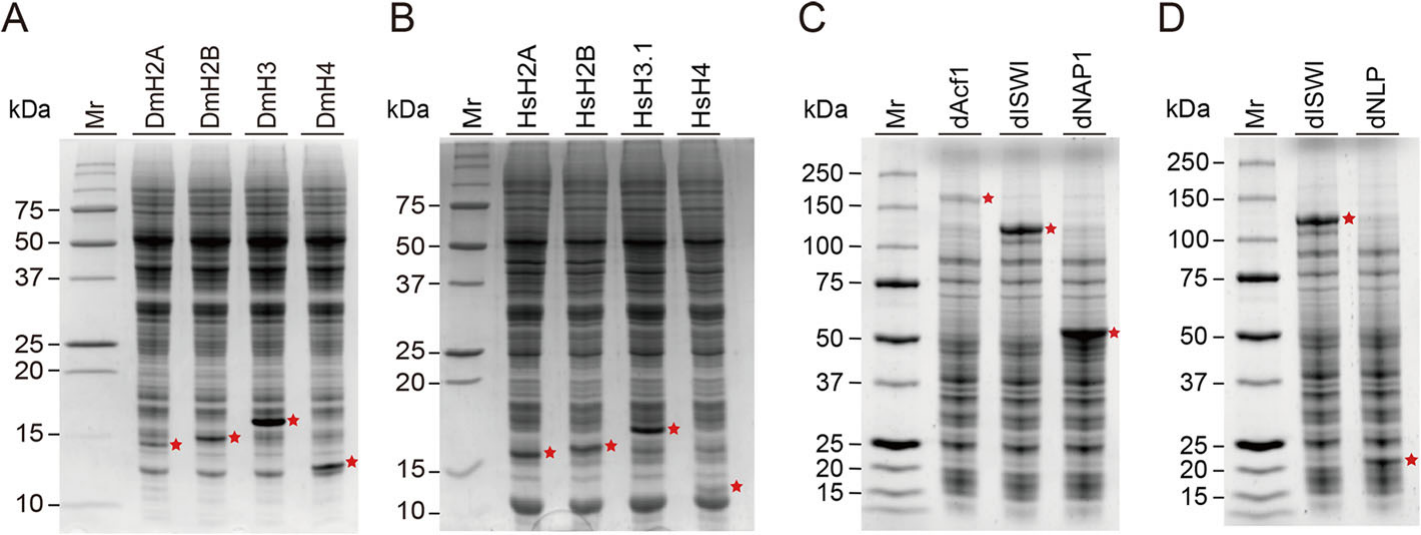
*Drosophila* and human core histone proteins, and *Drosophila* chromatin assembly factors expressed by wheat germ cell-free translation. Total translation mixtures containing **a** *Drosophila* histones, **b** human histones, **c** *Drosophila* chromatin assembly factors used in the *Drosophila* chromatin assembly, and **d** human chromatin assembly were separated by SDS-PAGE and visualized by Coomassie Blue stain. Histones were run on 18% SDS-PAGE, and chromatin assembly factors were analyzed on 10% SDS-PAGE, respectively. The red filled asterisks denote the in vitro synthesized proteins. Mr: protein molecular weight marker

The *Drosophila* core histones DmH2A, DmH2B, DmH3, and DmH4, and chromatin assembly factors, dAcf1, dISWI, and dNAP1 were cloned into the pEU-E01-MCS expression vector from the original pET plasmids [26, 40]. The *Drosophila* nucleoplasmin, dNLP, coding gene was amplified from the cDNA library of adult *Drosophila* whole body, then cloned into the pEU-E01-MCS vector. The human core histones were amplified from the human cDNA library and cloned into a pEU-E01-LICNot expression vector [41]. The pEU vector used in this study has been optimized to maximize the efficiency of in vitro wheat germ-based cell-free protein synthesis [41, 42]. The applied vectors do not possess an affinity tag coding region since we aimed to achieve the physiological state of histones and a single-step nucleosome assembly without affinity purification. Additionally, we performed a proteomic analysis of the wheat germ extract in order to confirm that there is no detectable wheat histone peptide in the extract (see Additional file 1). In the total of 744 proteins identified in the wheat germ cell-free extract, the most abundant proteins were annotated as heat shock proteins, followed by the proteins that related to protein synthesis such as a translation elongation factor. There were no wheat core histones H2A, H2B, H3, nor H4 found in the wheat extract. Prior to performing in vitro chromatin assembly reaction, all target genes were transcribed in vitro, and each protein product was cell-free synthesized in a cup dialysis mode and analyzed on SDS-PAGE gel (Fig. 1). Total translation mixtures of *Drosophila* core histones (Fig. 1a), along with human counterparts (Fig. 1b), and *Drosophila* chromatin assembly factors, dACF1, dISWI, dNAP1, and dNLP (Fig. 1c and d) were successfully produced with their expected molecular weight (Table 1). Among synthesized *Drosophila* core histones, DmH2A and DmH2B were synthesized less effectively, ~ 70% than DmH3 and DmH4 under a bilayer method using the same amount of mRNAs. In contrary to *Drosophila* core histones, the human histones HsH2A, HsH2B, HsH3.1, and H4 were produced less effectively by the bilayer method, ~ 20% of corresponding *Drosophila* histones, thus human core histones were synthesized by the cup dialysis mode (Fig. 1b), which is a more robust way of wheat cell-free synthesis [31]. By densitometry analysis of the SDS-PAGE, HsH2A, HsH2B, and HsH3.1 seemed to be synthesized equally, however, the product of HsH4 was ~ 30% less than other core histones. The further use of the wheat cell-free protein synthesis for chromatin assembly method was convincing based on the protein expression profiles.

**Table 1:** Data and properties of expressed proteins

Name	Gene	Uniprot ID	MW (kDa)	AA^a^	Source	References
DmH2A	*His2A*	P84051	13.4	124	*D. melanogaster*	[26]
DmH2B	*His2B*	P02283	13.7	123	*D. melanogaster*	[15]
DmH3	*His3*	P02299	15.4	136	*D. melanogaster*	[15]
DmH4	*His4*	P84040	11.4	103	*D. melanogaster*	[15]
HsH2A	*HIST2H2AA3*	Q6FI13	14.1	130	*H. sapiens* (Jurkat cell line)	*This study*
HsH2B	*H2BC4*	P62807	13.9	126	*H. sapiens* (Jurkat cell line)	*This study*
HsH3.1	*H3C1*	P68431	15.4	136	*H. sapiens* (Jurkat cell line)	*This study*
HsH4	*H4C1*	P62805	11.4	103	*H. sapiens* (Jurkat cell line)	*This study*
dAcf1	*Acf*	Q9V9T4	170.4	1476	*D. melanogaster*	[15]
dISWI	*Iswi*	Q24368	118.9	1027	*D. melanogaster*	[15]
dNAP1	*Nap1*	Q9W1G7	42.8	370	*D. melanogaster*	[15]
dNLP	*Nlp*	Q27415	17.0	152	*D. melanogaster*	[26, 29]
DmH1	*His1*	P02255	26.4	256	*D. melanogaster*	[29]

^a^Number of amino acids in each protein includes “Met” at the N-terminus

### Assembly of *Drosophila* chromatin using wheat germ cell-free synthesis

**Fig. 2:**
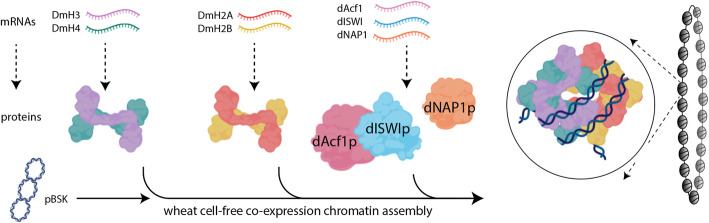
Flowchart of in vitro wheat germ cell-free chromatin assembly. The *Drosophila* in vitro wheat germ chromatin reconstitution method described in this study is shown. A pair of mRNAs encoding DmH3 and DmH4, DmH2A and DmH2B, and chromatin assembly factors, dAcf1, dISWI, and dNAP1, were co-translated in the presence of circular plasmid, and obtained product is circular closed chromatin. The image was created with the ADOBE Illustrator and BioRender.com

**Fig. 3:**
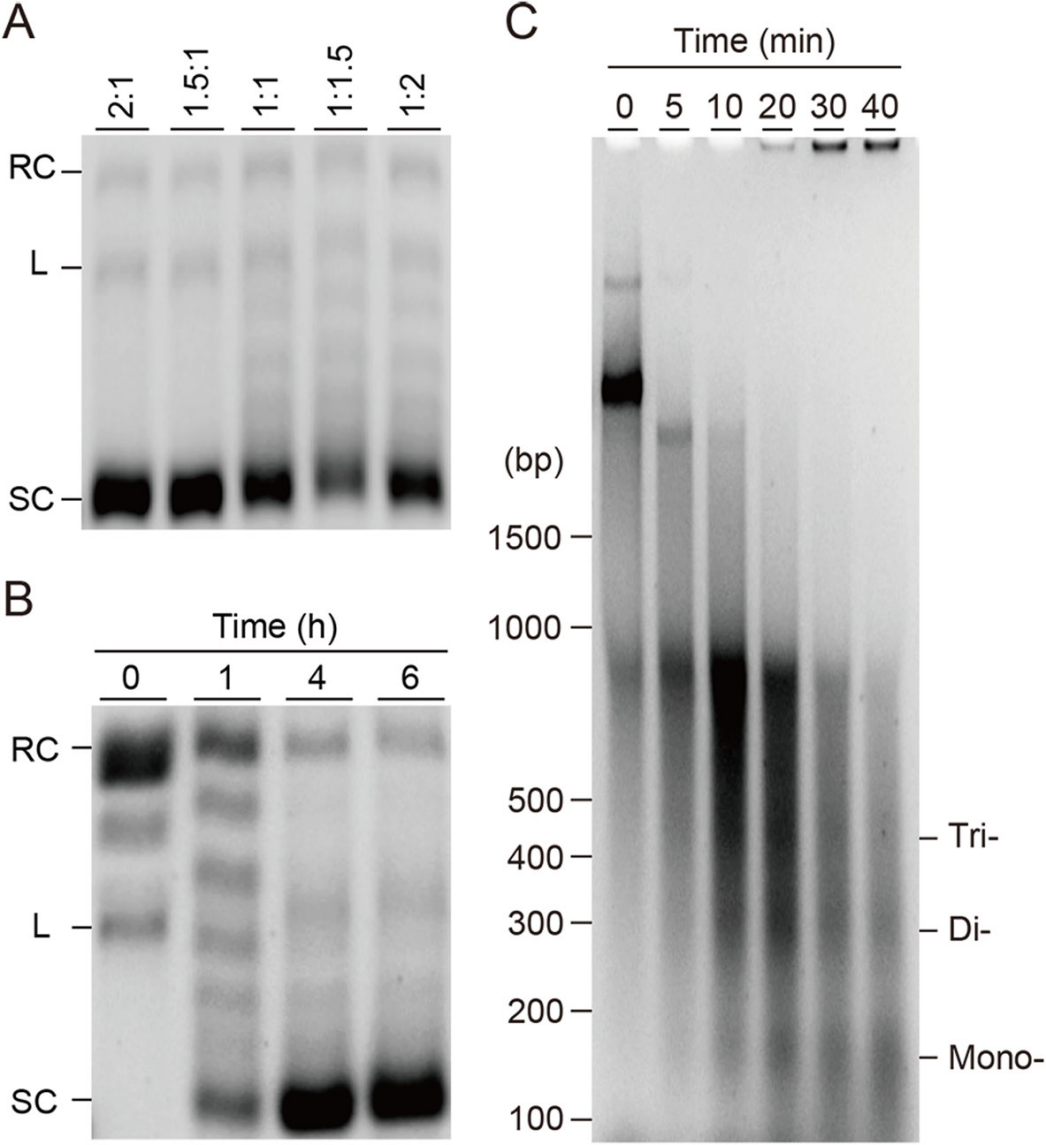
DNA supercoiling and MNase assays of *Drosophila* chromatin assembly reactions. **a** Determination of the appropriate ratio of DmH2A/DmH2B and DmH3/DmH4 mRNAs in the Drosophila chromatin reconstitution reaction. The incubation time was 4 h. Supercoiling assay was performed, samples were analyzed by 0.8% agarose TBE gel. Relaxed, linear, and supercoiled plasmid DNAs are indicated as RC, L, and SC, respectively. **b** Supercoiling assay of assembled *Drosophila* chromatin under the indicated reaction time 0, 1, 4, and 6 h, where 0 indicated the reconstitution reaction in the absence of mRNAs encoding histones. Supercoiling assay was performed, samples were analyzed by 0.8% agarose TBE gel. **c** Partial MNase digestion was performed on assembled *Drosophila* chromatin for the indicated time (0, 5, 10, 20, 30, and 40 min), then samples were run on 2.0% agarose gel. Bands corresponding to digestion fragment of the mono-, di-, and trinucleosomes were detected and indicated. Agarose gels were visualized by ethidium bromide and UV light. Mr: DNA molecular weight marker

Generally, *Drosophila* chromatin assembly methods employ either bacterially expressed and refolded histones or native histones extracted from eukaryotic cell nuclei, along with the ATP-dependent chromatin remodeling factor and nucleosome assembly factor in the presence of template DNA [23, 43, 44]. To develop a new chromatin assembly method by using wheat germ cell-free translation, initially, we aimed to reconstitute *Drosophila* chromatin with the reported ATP-dependent chromatin remodeling factors and nucleosome assembly chaperone, dAcf1, dISWI, and dNAP1 [25, 26]. The scheme of our novel single-step chromatin assembly method is shown in Fig. 2, which describes the in vitro co-expression of proteins, and the reconstitution of chromatin can be accomplished concurrently in one tube. We hypothesized, if histones and chromatin assembly factors are synthesized successfully, translation and assembly may occur concomitantly. To perform the co-expression chromatin assembly in the wheat cell-free system, a set of the pre-transcribed mRNAs encoding four core histones DmH2A, DmH2B, DmH3, and DmH4 were co-translated with the mRNAs of *Drosophila* chromatin assembly factors, dAcf1, dISWI, and dNAP1 in the presence of carrier DNA with unbiased sequence, pBSK plasmid and a trace amount of topoisomerase I enzyme under a bilayer mode. For ease, the two premixed mRNAs encoding core histones were prepared by pairwise, DmH2A/DmH2B, and DmH3/DmH4 for translation-coupled chromatin assembly reaction. The ratio of mRNAs of DmH2A/DmH2B and DmH3/DmH4 was optimized in order to make equimolar proteins among the histone octamers during the reaction. According to the earlier studies, the in vitro *Drosophila* chromatin assembly may be completed in 2 to 4 h [26, 45]. Thus, a series of different ratio of mRNAs encoding DmH2A/DmH2B and DmH3/DmH4 was tested that range from 2:1, 1.5:1, 1:1, 1:1.5, and 1:2, and incubated for 4 h at 26 °C. Importantly, the amount of expected histone protein products to the DNA template in this reaction condition was adjusted to be around 1:1 (w/w) mass ratio by estimating the histone synthesis for 4 h. Subsequently, the formation of circular chromatin was verified by DNA supercoiling assay (Fig. 3a and Fig. S1a), which is a standard method to evaluate chromatin assembly on closed circular DNA [46, 47]. Under the tested assembly condition, the 1.5:1 and 2:1 ratio of DmH2A/DmH2B to DmH3/DmH4 resulted in the highest degree of supercoil formation, consistent with the observation of independent histone protein synthesis shown in Fig. 1a. The following experiments were performed by applying the 1.5:1 ratio of the mRNAs (DmH2A/DmH2B:DmH3/DmH4). Next, we aimed to test the reaction time as 1, 4, and 6 h, followed by supercoiling assay (Fig. 3b and Fig. S1b). We concluded that *Drosophila* chromatin was sufficiently reconstituted after 4 h and unchanged after prolonged reaction time. To test whether the assembled chromatin is canonical DNA-histone octamer complex or not, in other words, if DNA is wrapped around histone octamers for the expected length, we assessed the apparent nucleosome repeat length (NRL) by MNase assay (Fig. 3c). The formation of mono-, di-, and trinucleosomes was observed after 5 min of MNase digestion, and the domination of mononucleosomal DNA was detected under longer MNase digestion. The average NRL of rigorous digestion by MNase was estimated at 152 ± 3 bp, in agreement with the reported nucleosomal DNA of around 145–147 bp [3, 4, 45]. From these results, the *Drosophila* chromatin was assembled in the system of co-expression wheat cell-free synthesis, proven by the supercoiling formation with the canonical nucleosome unit, an octamer wrapped around 1.7 times by approximately 150 bp DNA.

### Human chromatin assembly using wheat germ cell-free synthesis

**Fig. 4:**
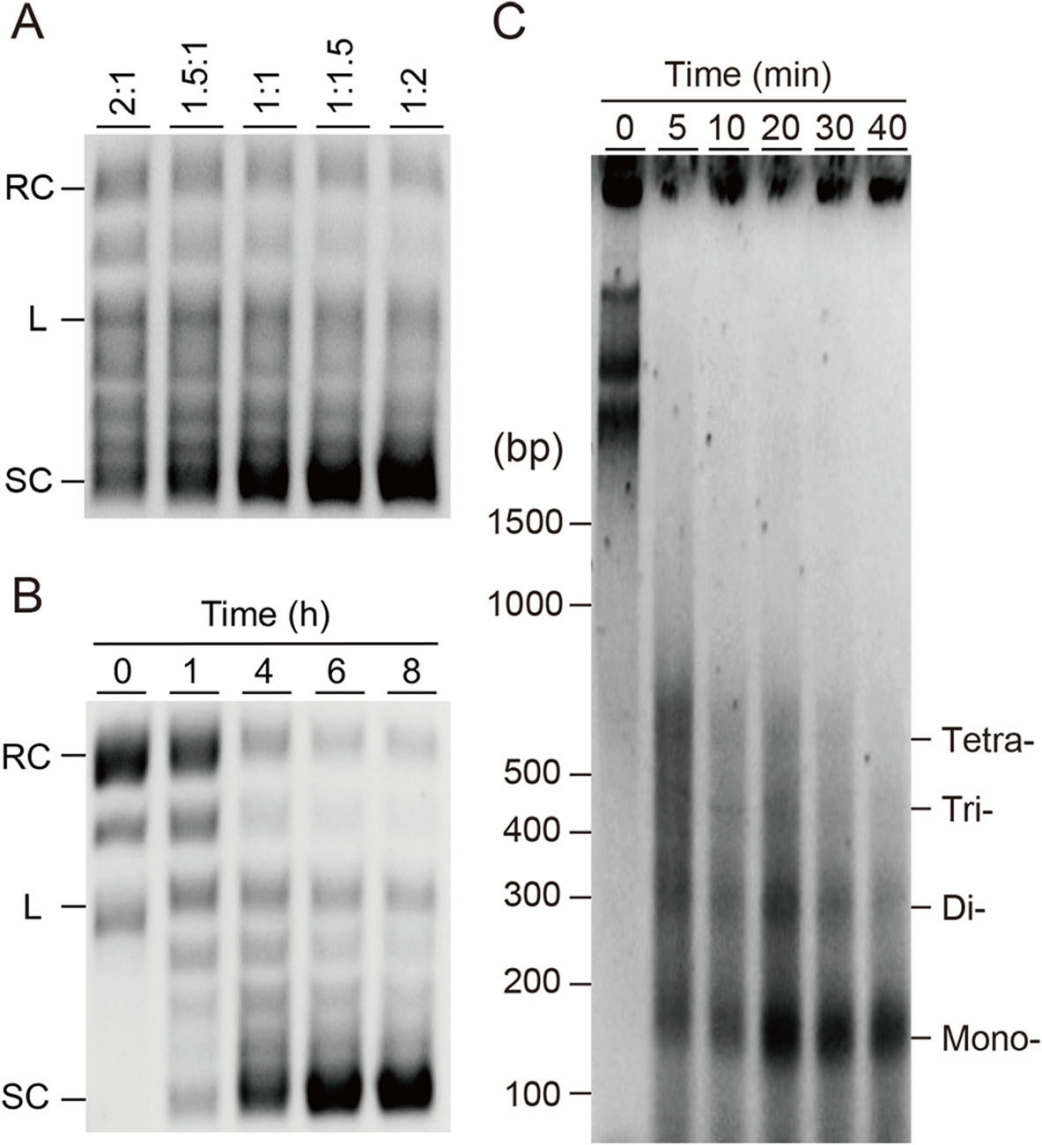
Optimization and confirmation of human chromatin assembly reaction. **a** DNA supercoiling assay to optimize the ratio of HsH2A/HsH2B and HsH3.1/HsH4 mRNAs in the chromatin reconstitution reaction for 4 h. **b** Supercoiling assay of assembled human chromatin. Incubation time indicated with 0, 1, 4, 6, and 8 h, where 0 indicated the reconstitution reaction in the absence of mRNAs encoding histones. Supercoils were analyzed by 0.8% agarose TBE gel. Relaxed, linear, and supercoiled DNA are indicated as RC, L, and SC, respectively. **c** Partial MNase digestion of assembled human chromatin, then samples were run on 2.0% agarose gel. Reconstituted chromatin was digested by MNase for the indicated time (0, 5, 10, 20, 30, and 40 min), and bands corresponding to digestion fragment of the mono-, di-, tri- and tetranucleosome were indicated. Mr: DNA molecular weight marker

Following the success of the *Drosophila* in vitro chromatin assembly, the reconstitution of human chromatin was tested in a co-expression manner in the presence of appropriate chromatin assembly factors and pBSK plasmid. Despite a long history of in vitro chromatin reconstitution of the *Drosophila* system, the human chromatin reconstitution had been somewhat challenging until recently. A recent report described the details of in vitro human chromatin assembly [29], of which they utilized dISWI and dNLP as essential chromatin assembly factors. Thus, we carried out the reconstitution of human chromatin by the in vitro co-expression of human core histones and *Drosophila* dISWI and dNLP in a bilayer mode. Analogous to the *Drosophila* chromatin assembly reaction for 4 h at 26 °C, the ratio of HsH2A/HsH2B to HsH3.1/HsH4 was first assessed by the best degree of supercoiling formation (Fig. 4a and Fig. S2a), and an optimum mRNA ratio between HsH2A/HsH2B and HsH3.1/HsH4 was determined to be 1:1.5 and 1:2. Under the optimized histone mRNA ratio of 1:2 (HsH2A/HsH2B:HsH3.1/HsH4), the reconstitution reaction time was tested for 1, 4, 6, and 8 h followed by supercoiling assay (Fig. 4b and Fig. S2b). From this result, apparent complete supercoiling was obtained after 6 h, which is longer than the reported incubation time [29]. In the previous study, human chromatin was reconstituted by a two-step method, an initial pre-reconstitution of histone octamers extracted from the HeLa cells with dNLP followed by an ATP-dependent enzymatic core histone assembly on DNA. Hence, the current one-step co-expression chromatin assembly method is different from the reported reaction scheme, and we rationalize that this difference may affect reaction time. The in vitro reconstituted human chromatin was analyzed by MNase assay, and mono-, di-, tri-, and tetranucleosomes were obtained under a series of digestion time (Fig. 4c). The average NRL of human chromatin was assessed at around 152 ± 7 bp, suggesting that the newly assembled human chromatin in a co-expression protocol indeed consistent with the canonical nucleosome unit [29].

### Co-expression of four core histones and linker histone H1 assembly

**Fig. 5:**
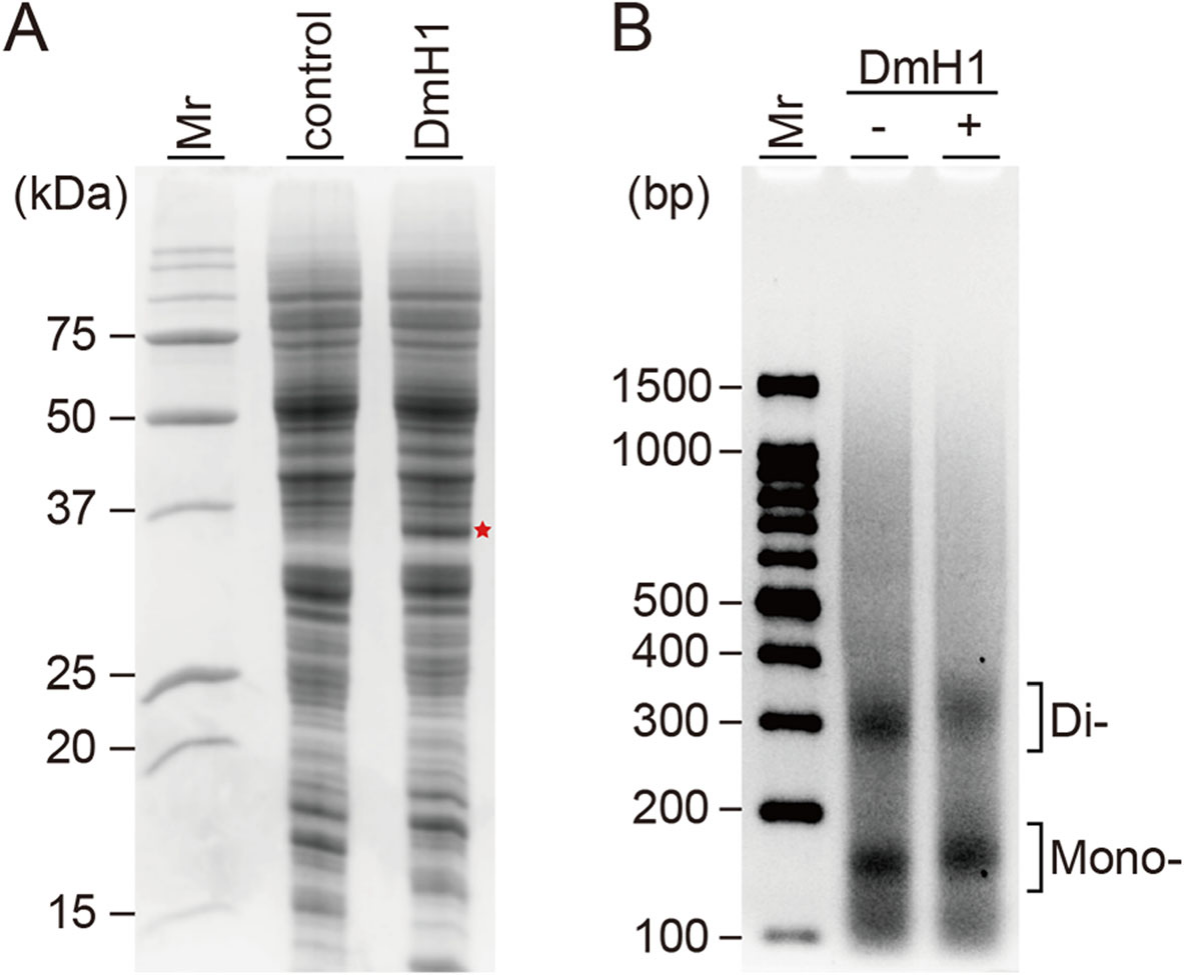
Co-expression *Drosophila* chromatin assembly in the presence of DmH1 mRNA **a** Production of DmH1 was analyzed by 18% SDS-PAGE and visualized by Coomassie Blue stain. Mr: protein molecular weight marker. **b** MNase assay was performed for 20 min at 25 °C on assembled *Drosophila* chromatins prepared in the absence (−) and presence (+) of DmH1 mRNA in the co-expression reaction. MNase digestion was performed right after chromatin assembly for the indicated time, then samples were run on 2.0% agarose gel. Mr: DNA molecular weight marker

To provide evidence that the reconstituted chromatin can be used for further downstream applications, we had assessed whether the linker histone H1 could be assembled into *Drosophila* chromatin. DmH1 was amplified from *Drosophila* cDNA, cloned, in vitro transcribed, then in vitro translated (Fig. 5a). Co-expression *Drosophila* chromatin assembly was performed in the presence of the premixed mRNAs of four core histones, three chromatin assembly factors, and DmH1-coding mRNA. The NRL of reconstituted chromatins was assessed by MNase assay in the absence and the presence of DmH1 (Fig. 5b). A time-dependent MNase digestion of the histone DmH1 associated, reconstituted chromatin can be seen in Supplementary Figure 4. Apparent NRL of *Drosophila* chromatin with DmH1 was estimated to be 175 ± 2 bp, which is approximately 23 bp longer than the chromatin without DmH1 (Fig. 3c). Thus, the linker histone H1 incorporation was thought to be achieved in this method.

## Discussion

In this study, both *Drosophila* and human core histones, and the protein factors that are essential to the chromatin assembly in vitro were successfully synthesized by the wheat cell-free protein translation (Fig. 1). As was shown in a framework of our method in Fig. 2, both *Drosophila* and human chromatins were in vitro reconstituted (Figs. 3 and 4). Although we developed a new method to reconstitute both *Drosophila* and human chromatins in a co-expression manner, there are distinct differences between the two systems. Between the two systems, different ratios of a pair of H2A/H2B and H3/H4 mRNAs are needed to be used in the reactions, and also reconstitution time was longer in the human chromatin than the *Drosophila* chromatin. We initially assumed that the two systems are comparable since the amino acid sequences of histone proteins are highly conserved between two species. However, the amount of core histone products between *Drosophila* and human by the wheat germ cell-free synthesis are quite different (Fig. 1). Moreover, the productivities among core histones were different from each other, i.e., more DmH3/DmH4 were produced than the DmH2A/DmH2B; more HsH2A/HsH2B/HsH3.1 were synthesized than the HsH4. Although, in general, the wheat germ cell-free protein synthesis does not require codon optimization, our result indicated that the codon usage of mRNAs encoding *Drosophila* and human histones affects the wheat protein synthesis rate [48]. Consistent with this notion, different ratios of two pairs of histones H2A/H2B and H3/H4 mRNAs were optimized between *Drosophila* and human chromatin assembly reactions. The *Drosophila* chromatin assembly was optimized at around 1.5:1 to 2:1 (DmH2A/DmH2B:DmH3/DmH4) ratio (Fig. 3a), while the ratio was 1:1.5 to 1:2 (HsH2A/HsH2B:HsH3.1/HsH4) for human (Fig. 4a). We concluded that the differences found between *Drosophila* and human chromatin assembly systems mainly result from the fitness of the codon-usages in the wheat cell-free protein synthesis platform.

Additionally, we speculated that the different chromatin assembly factors used in the *Drosophila* and human chromatin might affect the time of chromatin reconstitution. The *Drosophila* chromatin was reconstituted by using the well-documented chromatin assembly system, which employs a combination of the *Drosophila* ATP-utilizing assembly factor (dAcf1 and dISWI) and nucleosome assembly protein (dNAP1). Consistent with the previous studies, the *Drosophila* chromatin in our system was completed within 4 h. While, the combination of human core histones, *Drosophila* nucleoplasmin (dNLP), and dISWI was used in the human chromatin assembly, and the optimum reaction time was determined to be 6 h. Preeminently histone H3s and H4s, each of which differ only one amino acid between *Drosophila* and human (Fig. S3a and b), while the protein sequences between DmH2A and HsH2A of 84.6% similarity (110/130AA), and DmH2B and HsH2B of 78.6% similarity (99/126 AA) (Fig. S3c and d). Particularly, the first 30 amino acids from the N-terminus of DmH2B and HsH2B are highly variable, and only 9 amino acids are conserved (Fig. S3d). In the previous studies, an ortholog of dNLP in *Xenopus* oocytes, eNP, was shown to interact with *Xenopus* histone H2A/H2B dimer, H3/H4 dimer or tetramer, and octamer prior to the incorporation of octamers into nucleosomes, and this process was suggested to occur sequentially in the nuclei [49, 50]. Thus, we think that different histone sequences between *Drosophila* and human histones H2As and H2Bs affect the sequential formation of human histone octamer, and so nucleosome assembly mediated by dNLP. This finding is not a concern in the reported human nucleosome reconstitution method, which utilizes the dISWI and the pre-reconstituted dNLP-histone octamer complex, hence dNLP would only interact with the pre-reconstituted octamer before assembly [29].

Finally, we performed the chromatin and linker histone H1 co-assembly reaction to prove that the chromatins assembled in this study can be utilized for downstream applications. *Drosophila* chromatin was reconstituted in the presence of the premixed mRNAs encoding four core histones and linker histone, and other necessary factors used in the co-expression chromatin assembly reaction (Fig. 5). The assembled *Drosophila* chromatin in the presence of DmH1 showed longer NRLs than the chromatin without linker histone assessed by MNase assay (Fig. 5b and Fig. S4). Thus, the reconstituted chromatin is thought to be useful for downstream applications. Such downstream applications may involve epigenetic enzyme screening, including acetyltransferases, methyltransferases, and others, and ultimately in vitro reconstitution of the currently unavailable protein-chromatin complex such as heterochromatin reconstitution, can be achieved.

The most significant advantage of this method compared to other conventional chromatin assembly methods is that chromatin dynamics coop with other proteins/enzymes that can be reconstituted since the entire procedure is in one tube. In the conventional methods, a set of core histones is prepared before chromatin assembly reaction in either a salt dialysis method [16–18] or ATP-dependent chromatin assembly reactions [23–29]. In contrast, our method can provide a streamline of translation of core histones (and histone H1), chromatin assembly factors, and likely other chromatin-associated proteins in a reaction. Thus, unlike conventional methods, our method can be well suited to study chromatin dynamics.

## Conclusions

In this study, we report a novel in vitro co-expression chromatin assembly method by utilizing *Drosophila* and human chromatin systems (Figs. 1 and 2). All the histone proteins and chromatin assembly factors were expressed by in vitro wheat germ cell-free protein synthesis (Fig. 1). The mRNAs encoding *Drosophila* core histones, DmH2A, DmH2B, DmH3, and DmH4, and *Drosophila* chromatin assembly factors were co-translated with the circular plasmid DNA, the reaction was optimized, and assessed by DNA supercoiling and MNase assays (Fig. 3). Analogously, the four human core histones, HsH2A, HsH2B, HsH3.1, and HsH4, and *Drosophila* chromatin assembly factors, dISWI, and dNLP were co-translated and assembled on the circular plasmid, optimized, then subjected to supercoiling and MNase assays (Fig. 4). Several surprising differences were observed between *Drosophila* and human chromatin assembly reaction, such as the ratio of the input mRNAs encoding core histones and reconstitution time. We discussed multiple possibilities related to the amino acid sequences between *Drosophila* and human histones (Fig. S3) and the codon usages between two species that may explain the observed differences between the two systems. At last, histone H1-containing *Drosophila* chromatin was reconstituted successfully (Fig. 5), which indicates a potential usage for downstream applications [29].

Conventional methods typically carry out in vitro chromatin assembly in two-steps, which allows the equimolar formation of histone octamer prior to the assembly, thus the reconstitution reaction may only rely on the activity of ATP-dependent chromatin remodeling factor, and nucleosome assembly factors. In contrast, our method let protein synthesis and chromatin assembly concomitantly, which makes this method fundamentally distinct from conventional methods.

In summary, the described method presents a quick, convenient, and perhaps near-physiological tool for chromatin reconstitution of both *Drosophila* and human, providing a straightforward approach to future chromatin research, including chromatin formation, chromatin epigenetics, and epigenetic drug studies.

## Methods

### Materials

General reagents used in cDNA synthesis (Nippon Genetics), cloning (Toyobo), in vitro transcription (Promega), protein expression, including wheat germ extract (WEPRO7240H Lot: 15AQ02, CellFree Sciences) and SUB-AMIX translation buffer (CellFree Sciences), and creatine kinase (Roche Diagnostics, GmbH) were purchased. Reagents used in chromatin assembly reaction, including pBluescript II SK plasmid DNA (Addgene), SUB-AMIX solution (CellFree Sciences), creatine kinase (Roche Diagnostics, GmbH), and Topoisomerase I (Takara Bio Inc.) were also purchased. For gel analyses,100 bp DNA molecular weight marker (Toyobo) and protein size marker (Bio-Rad Laboratories) were purchased.

### cDNA library

Total RNA extracts of human culture Jurkat cell line (RCB3052, RIKEN BioResource Research Center) were a kind gift of Ryotah Uehara (Hokkaido University) and total RNA extracts of adult *Drosophila* were prepared by using FastGeneTM kit (Nippon Genetics). cDNA libraries were synthesized by using 100 ng total RNA and with random 6-mer primer with PrimeScript II (Takara Bio Inc) by following the manufacturer protocol.

### Cloning

Genes encoding *Drosophila* core histones, DmH2A (UniProt ID: P84051), DmH2B (UniProt ID: P02283), DmH3 (UniProt ID: P02299), and DmH4 (UniProt ID: P84040), linker histone H1 (UniProt ID: P02255) and *Drosophila* chromatin assembly factors dAcf1 (UniProt ID: Q9V9T4), dISWI (UniProt ID: Q24368), and dNAP1 (UniProt ID: Q9W1G7) were a kind gift from Prof. Kadonaga (UC San Diego, USA). Genes encoding human core histones HsH2A (UniProt ID: Q6FI13), HsH2B (UniProt ID: P62807), HsH3.1 (UniProt ID: P68431), and HsH4 (UniProt ID: P62805), and *Drosophila* chromatin assembly factors dNLP (UniProt ID: Q27415) were amplified from human and *Drosophila* cDNA libraries, respectively. Genes of *Drosophila* core histones and chromatin assembly factors were cloned into a pEU-E01-MCS expression vector (CellFree Sciences) by PIPE-cloning [51]. Genes of human core histones were inserted into the pEU-E01-LICNot vector by ligation-independent cloning [52]. The pEU-E01-LICNot, the affinity tag-free version of the vector was generated by the elimination of His_6_-tag by PCR mediated deletion [53] with the usage of the following primers 5′-TATTGGATTGGAAGTAACTAGTGATATCTTGGTGA-3′ and 5′- CCAAGATATCACTAGTTACTTCCAATCCAATATTG-3′.

### Proteomic analysis of the wheat germ cell-free extract

The sample preparation and proteomic analysis were performed by following the previous study [54]. Briefly, 10 μg equivalent of the wheat germ cell-free extract (WEPRO7240H Lot: 15AQ02, CellFree Sciences) was precipitated by 20% (vol/vol) trichloroacetate (Wako Pure Chemical Corporation), and incubated for 20 min on ice, followed by ice-cold acetone wash for two times. The pellet was resuspended in 10.0 μl of 8 M urea, then 70 μl 25 mM ammonium bicarbonate (Wako Pure Chemical Corporation) was added. The reduction and alkylation reactions were performed by adding 5 mM DTT (Wako Pure Chemical Corporation) for 30 min at 50 °C, and 15 mM iodoacetamide (Wako Pure Chemical Corporation) for 30 min at 25 °C in the dark, respectively. Tryptic digestion was performed by adding 0.5 μg of proteomic grade trypsin (Roche Diagnostics, GmbH), and incubated for 4 h at 37 °C. The trypsin-digested peptides were separated by using a nanoLC1000 (Thermofisher Scientific) hooked up with a C18 column (NTCC-360/75–3-125; Nikkyo Technos) connected to a hybrid linear ion trap-orbitrap mass spectrometer (Q-Exactive Plus; ThermoFisher Scientific) and operated by using an Xcalibur software v3.1 (Thermofisher Scientific). Peptide fragments were separated by a linear gradient rased from 5 to 30% acetonitrile in 0.1% formic acid (Fisher Chemical) for 240 min and monitored at a scan range of 300.0 to 20,000 m/z and a resolution of 70,000. Raw MS/MS data were analyzed by using Proteome Discoverer software v1.4 (ThermoFisher Scientific) under the following settings: the peptide mass tolerance was set to 10.0 ppm, and the fragment mass tolerance was set to 0.8 Da. A list of proteins encoded in the *Triticum aestivum* genome was obtained from the NCBI on April 2014. Fixed modification of carbamidomethyl at cysteine and dynamic modifications of oxidation at methionine were used for the database search. The abundance of each protein was estimated by the Sequest score calculated by the Proteome Discoverer software.

### In vitro transcription and translation

The plasmid constructs were transcribed by in vitro transcription according to the previous study [30, 33], briefly, 2.0 μg vector was transcribed for 4 h at 37 °C in 20.0 μl total reaction volume. 6 U RNase-free DNase I (Nippon Gene) was added to remove the plasmid DNA templates. The mRNAs were purified by acidic phenol-chloroform-IAA (pH 4.5) extraction followed by ethanol precipitation, resuspended in 20.0 μl of RNase-free water. The amount of mRNA’s was determined by spectrophotometer at 260 nm. All protein products shown in Fig. 1 and Fig. 5a were translated by a cup dialysis method [31, 42] by mixing 20.0 μl purified individual mRNA, 16.7 μl wheat germ extract, 4.0 μg creatine kinase, and 48.0 μl 1x SUB-AMIX solution in a total of 100.0 μl reaction in the dialysis cup (10 K MWCO, Slide-A-Lyzer, Thermofisher Scientific). The mixture was incubated for 72 h at 15 °C with 1.5 ml 1x SUB-AMIX filling the outside of the dialysis cup. Both bilayer and dialysis cup methods yielded approximately 5–10.0 μg target protein per reaction. Then, 1.5 μl each of translation products was mixed with 2x loading buffer (Bio-Rad Laboratories), incubated for 5 min at 95 °C, then analyzed by SDS-PAGE. 18.0% SDS-PAGE gel and Coomassie Blue staining were used for *Drosophila* and human histone proteins, 10% stain-free SDS-PAGE (Bio-Rad Laboratories) was used for chromatin assembly factors and proteins were visualized by the gel documentation system (Bio-Rad Laboratories). For DmH1, a sample was run on 18% SDS-PAGE followed by Coomassie Blue staining. Expected molecular weights of in vitro translated proteins: *Drosophila* core histone DmH2A 13.4 kDa, DmH2B 13.7 kDa, DmH3 15.6 kDa, DmH4 11.4 kDa; human core histone HsH2A 14.1 kDa, HsH2B 14.0 kDa, HsH3.1 15.4 kDa, HsH4 11.4 kDa; *Drosophila* chromatin assembly factors, dAcf1 170.4 kDa, dISWI 118.9 kDa, dNAP1 42.8 kDa, dNLP 17.0 kDa (Table 1). The size of DmH1 on SDS-PAGE was larger than the expected molecular weight of 26.4 kDa, but our observed was consistent with the previous study [29].

### In vitro wheat germ cell-free nucleosome assembly of Drosophila chromatin

The mRNAs of *Drosophila* core histone DmH2A, DmH2B, DmH3 and DmH4 were mixed pairwise (DmH2A with DmH2B, DmH3 with DmH4) after in vitro transcription, precipitated and resuspend in 10.0 μl of RNase-free water (Nippon Gene). The ratio of appropriate amount core histone mRNAs was determined by testing the ratio of DmH2A/DmH2B and DmH3/DmH4 coding mRNAs from 1:0.5 to 1:2 in total of approximately 2.0 μg/reaction, then a stock mRNA mixture was made, that was used directly for chromatin assembly reaction. The reaction was performed by mixing 1.0 μl of *Drosophila* core histone mRNA mixture, 0.4 μl dAcf1, 0.4 μl dISWI, 0.4 μl dNAP1 mRNAs, 2.5 μg of supercoiled pBSK plasmid, 2 U of Topoisomerase I, 5.0 μl wheat germ extract, and 0.4 μg of creatine kinase and supplemented to 10.7 μl final volume with 1x SUB-AMIX solution. The mixture was gently underlaid to 103.0 μl of 1x SUB-AMIX solution in a 96-well plate to form bilayer. The reaction was carried out at the indicated time (Fig. 3) at 26 °C. The co-expression reaction of DmH1-containing chromatin assembly was performed by using the ratio of DmH2A/DmH2B:DmH3/DmH4:DmH1 = 1:0.67:0.42 under the same reaction conditions.

### In vitro wheat germ cell-free nucleosome assembly of human chromatin

The stock solution of the mRNAs of human core histone HsH2A, HsH2B, HsH3.1 and HsH4 was prepared pairwise as was described in the case of *Drosophila* core histone mRNAs. The ratio of appropriate amount core histone mRNAs was determined by testing the ratio of HsH2A/HsH2B and HsH3.1/HsH4 coding mRNAs from 1:0.5 to 1: 2 in total of approximately 2.0 μg/reaction, then a stock mRNA mixture was made, and used directly for chromatin assembly reaction. The chromatin assembly reaction was performed by mixing 1.0 μl of human core histone mRNA mixtures, 0.4 μl ISWI, 0.4 μl NLP mRNAs, 2.5 μg of supercoiled pBSK plasmid, 2 U of Topoisomerase I, 5.0 μl wheat germ extract, and 0.4 μg of creatine kinase and supplemented to 10.7 μl final volume with 1x SUB-AMIX solution. The mixture was gently underlaid to 103.0 μl of 1x SUB-AMIX solution in a 96-well plate to form bilayer. The reaction was carried out at the indicated time at 26 °C (Fig. 4).

### Supercoiling assay

Fifty-five point zero microliter of the total chromatin assembly reaction mixture was purified by phenol-chloroform-IAA (25:24:1, v/v) extraction (pH 8.0) followed by ethanol precipitation, and the purified DNA was resuspended in HD buffer (25 mM HEPES, 1 mM DTT, pH 7.6) containing a trace amount of Ribonuclease a (Macherey-Nagel GmbH & Co.). Supercoiled plasmid DNAs were separated by 0.8% TBE agarose gel in 0.5x TBE buffer, visualized with ethidium bromide (Nippon Gene), and analyzed by gel documentation system (Bio-Rad Laboratories).

### Micrococcal nuclease assay

MNase assay was performed by the addition of Micrococcal Nuclease (Bio-Rad Laboratories) and MNase buffer (20 mM Tris-HCl (pH 8.0), 5 mM NaCl, 2.5 mM CaCl_2_) to 28.0 μl of total reaction mixture followed by 5, 10, 20, 30, 40 min incubation at 25 °C. The final concentration of MNase for *Drosophila* chromatin was 14 U/μl and for human chromatin was 10 U/μl. For the DmH1-containing chromatin, 13 U/μl MNase was used. The assay was halted by EGTA, and proteins were eliminated by phenol-chloroform-IAA extraction followed by ethanol precipitation. The purified DNA was resuspended in HD Buffer (25 mM HEPES, 1 mM DTT, pH 7.6) containing a trace amount of Ribonuclease A and analyzed by 2.0% TAE agarose gel in 1x TAE buffer. Gels containing *Drosophila* DNA were stained with GelRed (Wako Pure Chemical Corporation) and human DNA was stained with ethidium bromide. The average mononucleosomal DNA length measurement was performed by the gel documentation system (Bio-Rad Laboratories).

## Supplementary Information

**Additional file 1:** **Supplementary Table S1.** Protein list of mass spectrometry analysis of the wheat germ extract. Results of proteins determined in the wheat germ cell-free extract (WEPRO7240H). Seven hundred forty-four proteins were identified in the total of 61,984 protein sequences listed in the FASTA file. The score indicated the enrichment of proteins calculated by the Proteome Discoverer.

**Additional file 2:** **Supplementary Figure S1.** Full scans of gel images shown in Fig. 3a and b. **a** A full gel image of determination of the appropriate ratio of DmH2A/DmH2B and DmH3/DmH4 mRNAs in the *Drosophila* chromatin reconstitution reaction shown in Fig. 3a. Supercoiling assay was performed, samples were analyzed by 0.8% agarose TBE gel. Relaxed, linear, and supercoiled plasmid DNAs are indicated as RC, L, and SC, respectively. **b** Supercoiling assay of assembled *Drosophila* chromatin under the indicated reaction time 0, 1, 4, and 6 h, where 0 indicated the reconstitution reaction in the absence of mRNAs encoding histones shown in Fig. 3b. Supercoiling assay was performed, samples were analyzed by 0.8% agarose TBE gel. Mr and pBSK indicate 1000 bp DNA molecular marker, and the supercoiled plasmid control, respectively. **Supplementary Figure S2.** Full scans of gel images shown in Fig. 4a and b. **a** A full gel image of DNA supercoiling assay to optimize the ratio of HsH2A/HsH2B and HsH3.1/HsH4 mRNAs in the chromatin reconstitution reaction for 4 h shown in Fig. 4a. **b** Supercoiling assay of assembled human chromatin. Incubation time indicated with 0, 1, 4, 6, and 8 h, where 0 indicated the reconstitution reaction in the absence of mRNAs encoding histones, which is shown in Fig. 4b. Supercoils were analyzed by 0.8% agarose TBE gel. Relaxed, linear, and supercoiled DNA are indicated as RC, L, and SC, respectively. Mr and pBSK indicate 1000 bp DNA molecular marker, and the supercoiled plasmid control, respectively. **Supplementary Figure S3.** Sequence alignment of *Drosophila* and human histones. Amino acid sequence alignment of **a** DmH3 and HsH3.1, **b** DmH4 and HsH4, **c** DmH2A and HsH3.1, **d** DmH2B and HsH2B are shown, respectively. Asterisks and light purple-highlighted amino acids showed the identical amino acid sequence between two species. **Supplementary Figure S4.** Time course of MNase digestion for the reconstituted *Drosophila* chromatosome. Assembled DmH1-incorporated chromatin was digested by 10 U/μl MNase for 0, 5, 10,20, 30, 40 min, then run on 2.0% agarose gel and visualized by ethidium bromide. Detected bands corresponding to mono-, di-, and tri-nucleosomes were indicated. NRL of reconstituted chromatosome was estimated to be 174.6 ± 2.1 bp.

## Data Availability

All data generated or analyzed during this study are included in this published article and in Supplementary Table S1, Supplementary Figure S1, and S2.

## Abbreviations

Acf: ATP-dependent chromatin assembly factor large subunit
IAA: Isoamyl alcohol
ISWI: Chromatin-remodeling complex ATPase chain Iswi
MNase: Micrococcal endonuclease
NAP1: Nucleosome assembly protein NAP-1
NLP: Nucleoplasmin-like protein
NRL: Nucleotide repeat length
PIPE: Polymerase Incomplete Primer Extension
d and Dm: *Drosophila melanogaster*
Hs: *Homo sapiens*
