# Supplementary material for: Reconstitution of Drosophila and human chromatins by wheat germ cell-free co-expression system
Source: BMC Biotechnol. 2020 Dec 1;20:62. doi: 10.1186/s12896-020-00655-6 (PMC7708258; doi:10.1186/s12896-020-00655-6)
Supplement: Supplementary file 2 — Additional file 2: Supplementary Figure S1. Full scans of gel images shown in Fig. 3a and b. a A full gel image of determination of the appropriate ratio of DmH2A/DmH2B and DmH3/DmH4 mRNAs in the Drosophila chromatin reconstitution reaction shown in Fig. 3a. Supercoiling assay was performed, samples were analyzed by 0.8% agarose TBE gel. Relaxed, linear, and supercoiled plasmid DNAs are indicated as RC, L, and SC, respectively. b Supercoiling assay of assembled Drosophila chromatin under the indicated reaction time 0, 1, 4, and 6 h, where 0 indicated the reconstitution reaction in the absence of mRNAs encoding histones shown in Fig. 3b. Supercoiling assay was performed, samples were analyzed by 0.8% agarose TBE gel. Mr and pBSK indicate 1000 bp DNA molecular marker, and the supercoiled plasmid control, respectively. Supplementary Figure S2. Full scans of gel images shown in Fig. 4a and b. a A full gel image of DNA supercoiling assay to optimize the ratio of HsH2A/HsH2B and HsH3.1/HsH4 mRNAs in the chromatin reconstitution reaction for 4 h shown in Fig. 4a. b Supercoiling assay of assembled human chromatin. Incubation time indicated with 0, 1, 4, 6, and 8 h, where 0 indicated the reconstitution reaction in the absence of mRNAs encoding histones, which is shown in Fig. 4b. Supercoils were analyzed by 0.8% agarose TBE gel. Relaxed, linear, and supercoiled DNA are indicated as RC, L, and SC, respectively. Mr and pBSK indicate 1000 bp DNA molecular marker, and the supercoiled plasmid control, respectively. Supplementary Figure S3. Sequence alignment of Drosophila and human histones. Amino acid sequence alignment of a DmH3 and HsH3.1, b DmH4 and HsH4, c DmH2A and HsH3.1, d DmH2B and HsH2B are shown, respectively. Asterisks and light purple-highlighted amino acids showed the identical amino acid sequence between two species. Supplementary Figure S4. Time course of MNase digestion for the reconstituted Drosophila chromatosome. Assembled DmH1-incorporated chromatin was diges [file 12896_2020_655_MOESM2_ESM.docx]

**
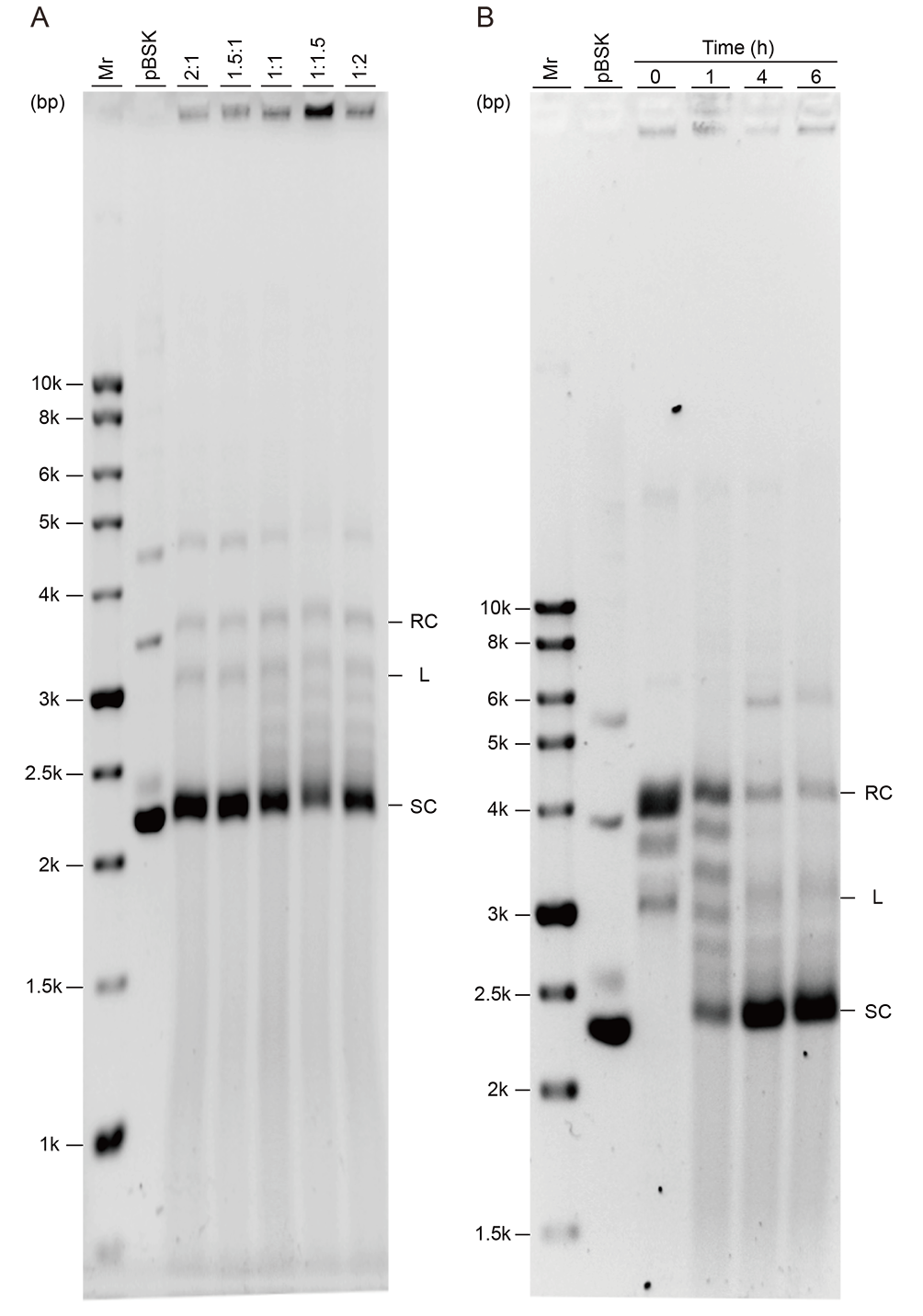
**

**Supplementary figure S1 : Full scans of gel images shown in Fig. 3A and B.**

**a** A full gel image of determination of the appropriate ratio of DmH2A/DmH2B and DmH3/DmH4 mRNAs in the *Drosophila* chromatin reconstitution reaction shown in Fig. 3A. Supercoiling assay was performed, samples were analyzed by 0.8% agarose TBE gel. Relaxed, linear, and supercoiled plasmid DNAs are indicated as RC, L, and SC, respectively. **b** Supercoiling assay of assembled *Drosophila* chromatin under the indicated reaction time 0, 1, 4, and 6 hours, where 0 indicated the reconstitution reaction in the absence of mRNAs encoding histones shown in Fig. 3B. Supercoiling assay was performed, samples were analyzed by 0.8% agarose TBE gel. Mr and pBSK indicate 1,000 bp DNA molecular marker, and the supercoiled plasmid control, respectively.

**
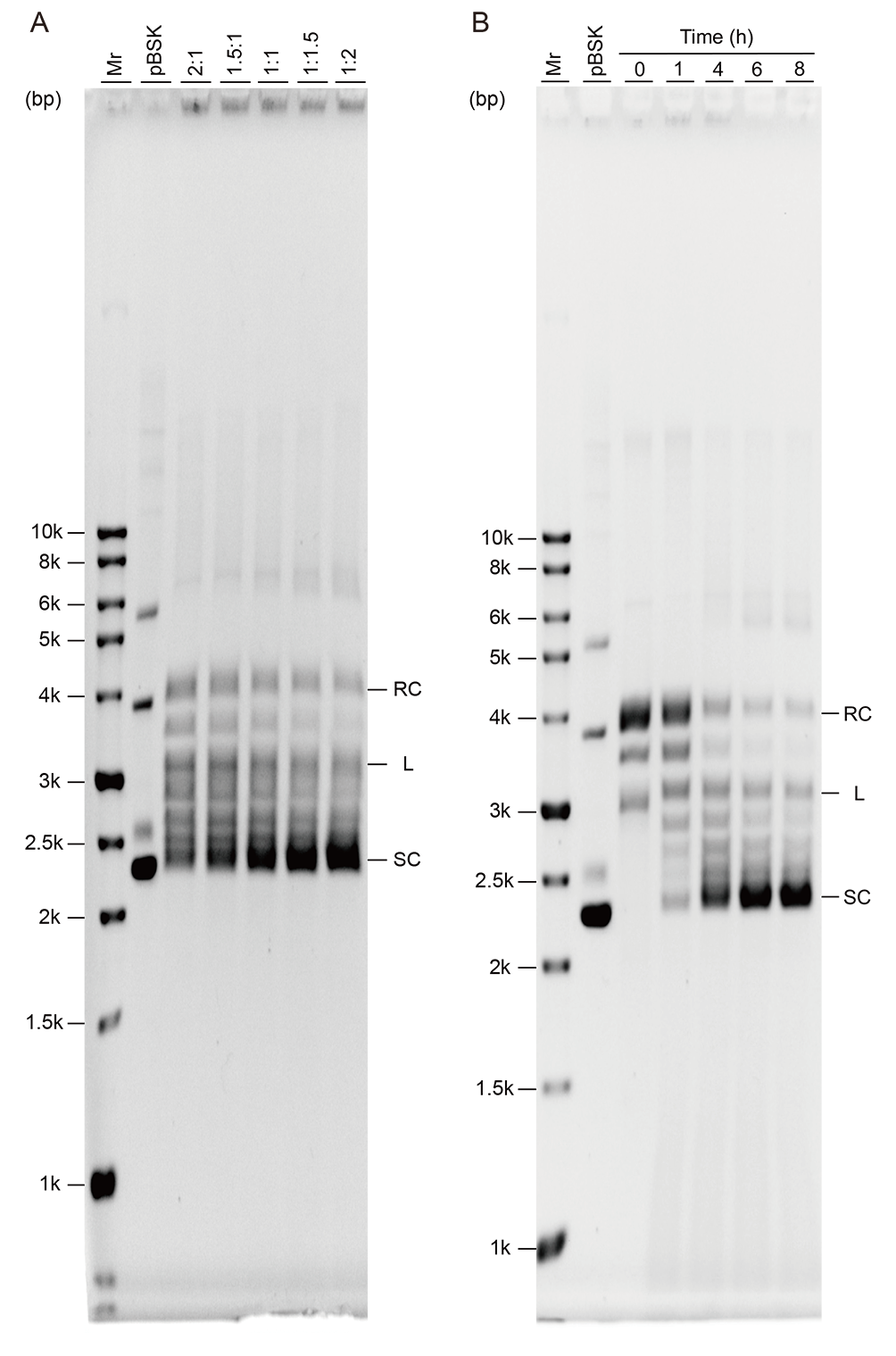
**

**Supplementary figure S2 : Full scans of gel images shown in Fig. 4A and 4B. a** A full gel image of DNA supercoiling assay to optimize the ratio of HsH2A/HsH2B and HsH3/HsH4 mRNAs in the chromatin reconstitution reaction for 4 hours shown in Fig. 4A. **b** Supercoiling assay of assembled human chromatin. Incubation time indicated with 0, 1, 4, 6, and 8 hours, where 0 indicated the reconstitution reaction in the absence of mRNAs encoding histones, which is shown in Fig. 4B. Supercoils were analyzed by 0.8% agarose TBE gel. Relaxed, linear, and supercoiled DNA are indicated as RC, L, and SC, respectively. Mr and pBSK indicate 1,000 bp DNA molecular marker, and the supercoiled plasmid control, respectively.


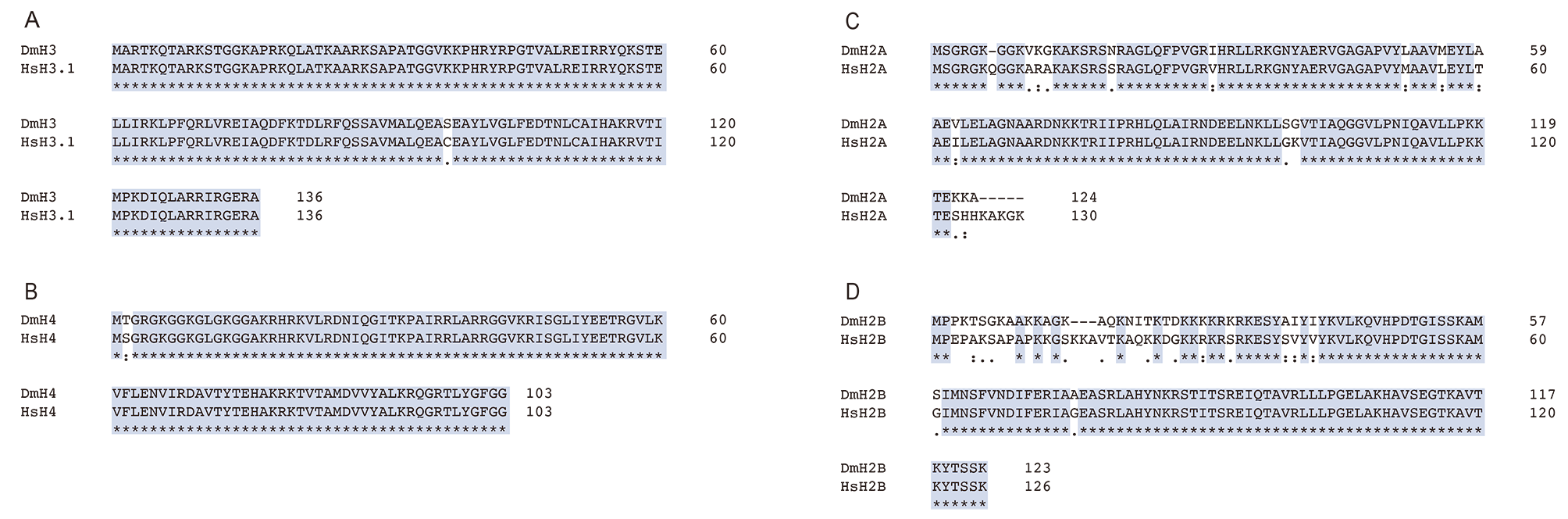


**Supplementary figure S3 : Sequence alignment of *Drosophila* and human histones.** Amino acid sequence alignment of **a** DmH3 and HsH3.1, **b** DmH4 and HsH4, **c** DmH2A and HsH3.1, **d** DmH2B and HsH2B are shown, respectively. Asterisks and light purple-highlighted amino acids showed the identical amino acid sequence between two species.


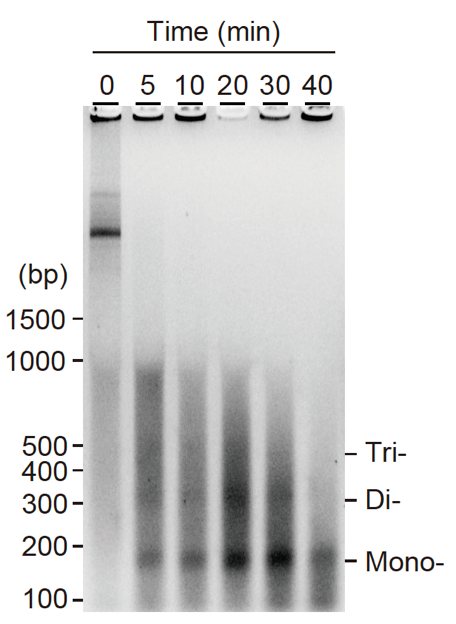


**Supplementary figure S4 : Time course of MNase digestion for the reconstituted *Drosophila* chromatosome.** Assembled DmH1-incorporated chromatin was digested by 10 U/μl MNase for 0, 5, 10,20, 30, 40 min, then run on 2.0% agarose gel and visualized by ethidium bromide. Detected bands corresponding to mono-, di-, and tri-nucleosomes were indicated. NRL of reconstituted chromatosome was estimated to be 174.6 ± 2.1 bp.
